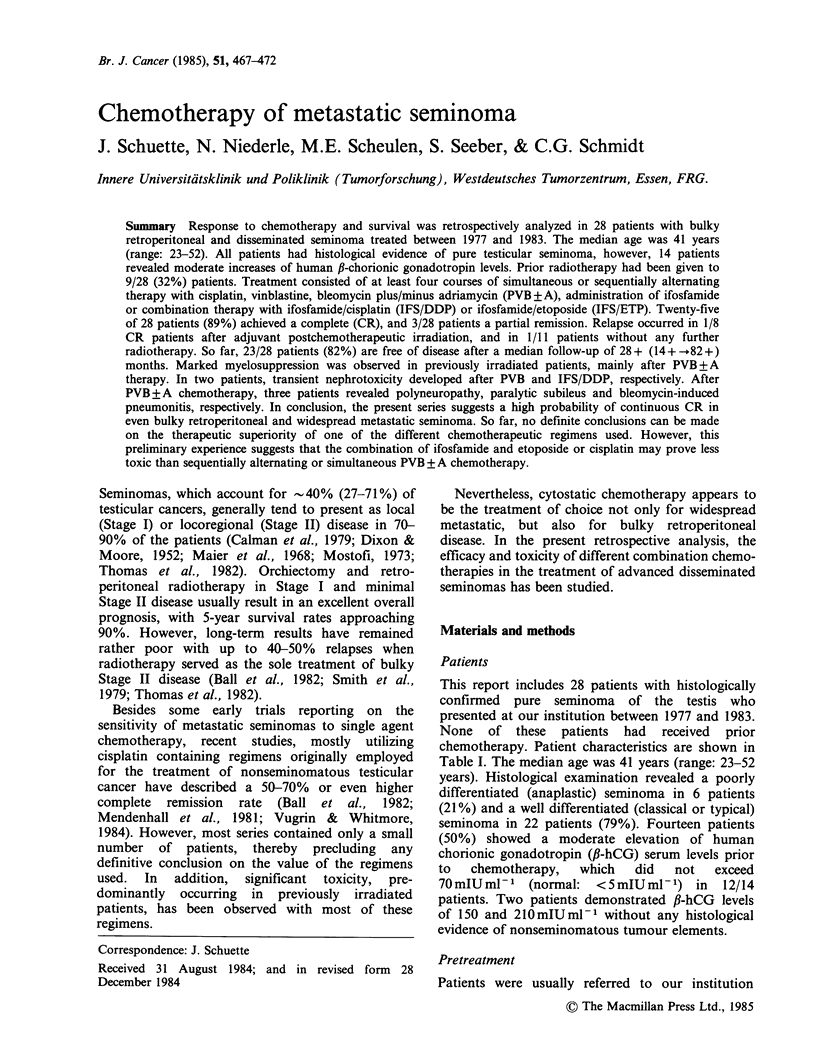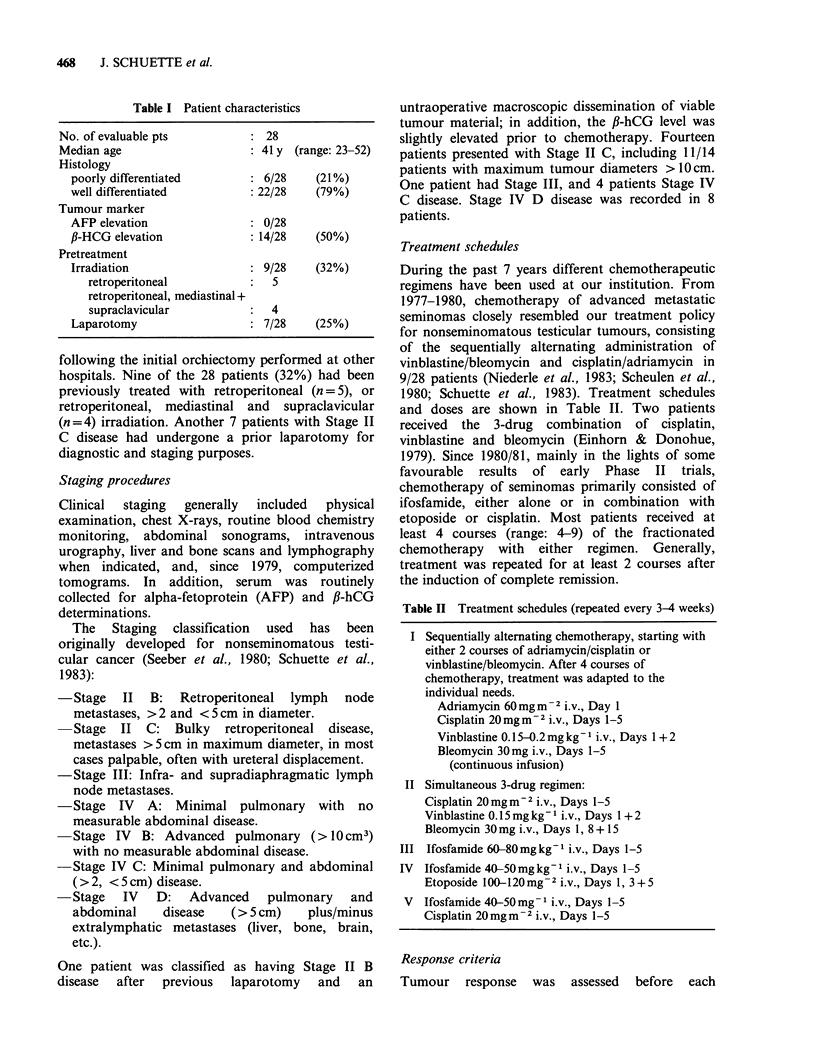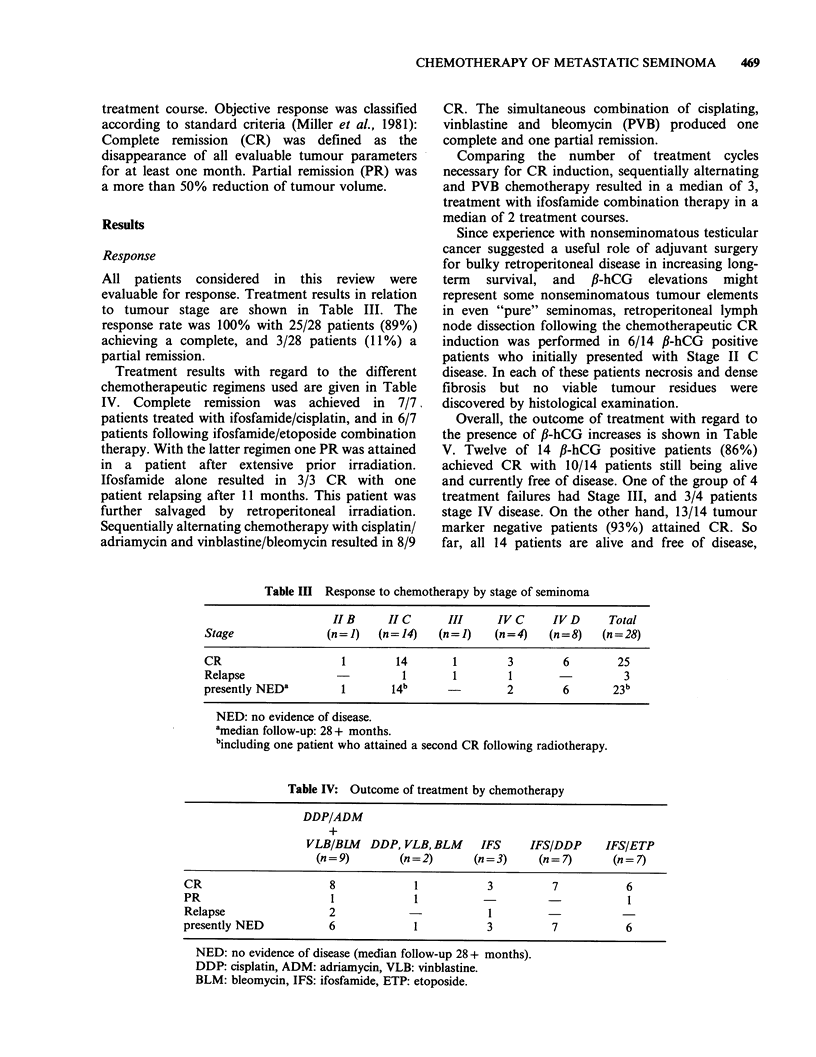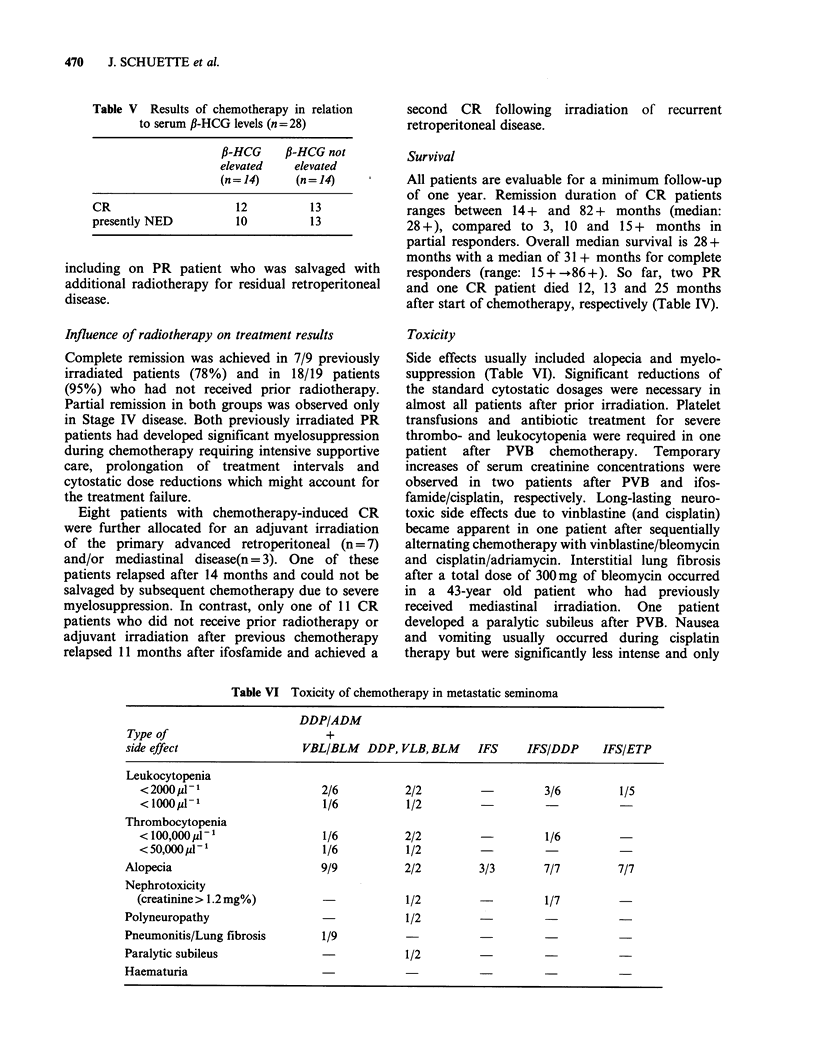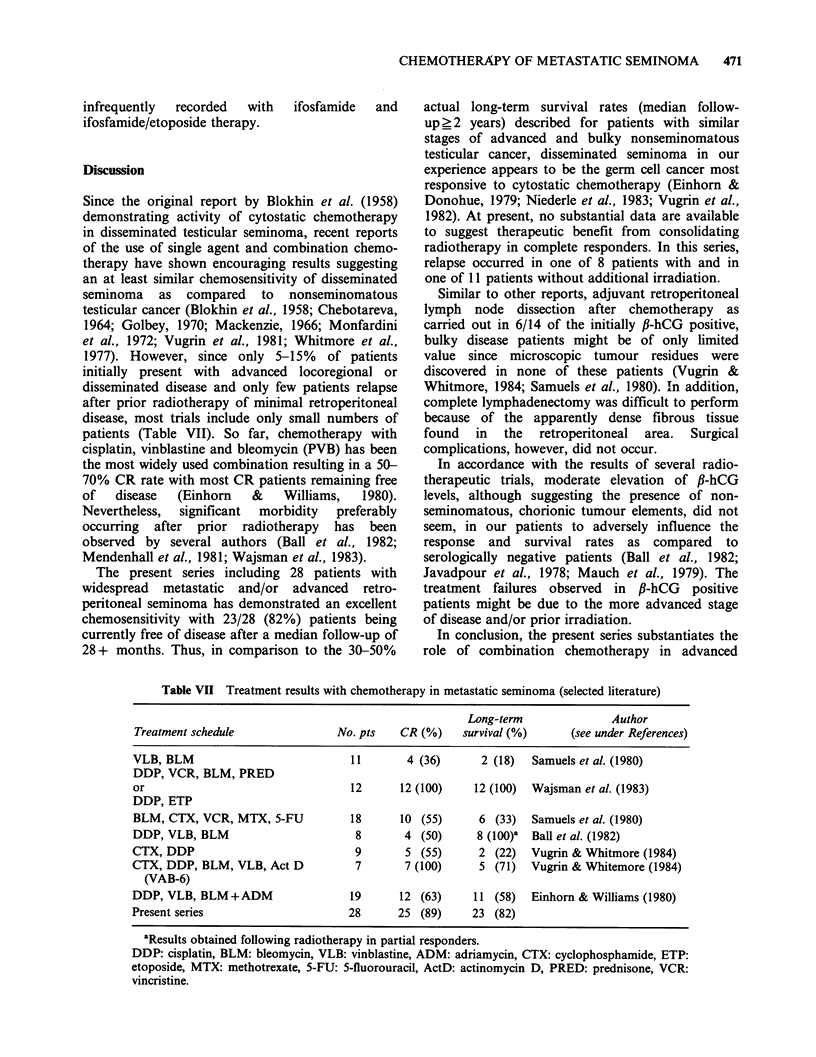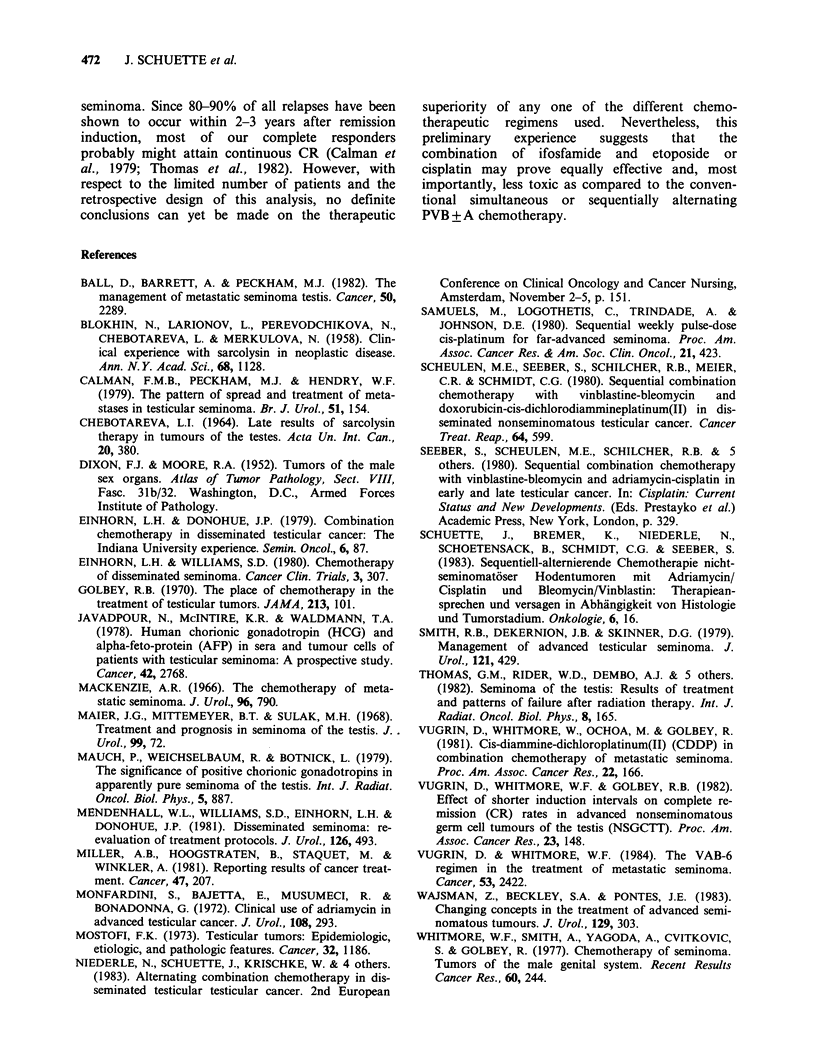# Chemotherapy of metastatic seminoma.

**DOI:** 10.1038/bjc.1985.67

**Published:** 1985-04

**Authors:** J. Schuette, N. Niederle, M. E. Scheulen, S. Seeber, C. G. Schmidt

## Abstract

Response to chemotherapy and survival was retrospectively analyzed in 28 patients with bulky retroperitoneal and disseminated seminoma treated between 1977 and 1983. The median age was 41 years (range: 23-52). All patients had histological evidence of pure testicular seminoma, however, 14 patients revealed moderate increases of human beta-chorionic gonadotropin levels. Prior radiotherapy had been given to 9/28 (32%) patients. Treatment consisted of at least four courses of simultaneous or sequentially alternating therapy with cisplatin, vinblastine, bleomycin plus/minus adriamycin (PVB +/- A), administration of ifosfamide or combination therapy with ifosfamide/cisplatin (IFS/DDP) or ifosfamide/etoposide (IFS/ETP). Twenty-five of 28 patients (89%) achieved a complete (CR), and 3/28 patients a partial remission. Relapse occurred in 1/8 CR patients after adjuvant postchemotherapeutic irradiation, and in 1/11 patients without any further radiotherapy. So far, 23/28 patients (82%) are free of disease after a median follow-up of 28+ (14+----82+) months. Marked myelosuppression was observed in previously irradiated patients, mainly after PVB +/- A therapy. In two patients, transient nephrotoxicity developed after PVB and IFS/DDP, respectively. After PVB +/- A chemotherapy, three patients revealed polyneuropathy, paralytic subileus and bleomycin-induced pneumonitis, respectively. In conclusion, the present series suggests a high probability of continuous CR in even bulky retroperitoneal and widespread metastatic seminoma. So far, no definite conclusions can be made on the therapeutic superiority of one of the different chemotherapeutic regimens used. However, this preliminary experience suggests that the combination of ifosfamide and etoposide or cisplatin may prove less toxic than sequentially alternating or simultaneous PVB +/- A chemotherapy.


*Br. J. Cancer* (1985), **51**, 467–472

### Chemotherapy of metastatic seminoma

J. Schuette, N. Niederle, M.E. Scheulen, S. Seeber, & C.G. Schmidt

*Innere Universitätsklinik und Poliklinik (Tumorforschung), Westdeutsches Tumorzentrum, Essen, FRG.*

**Summary** Response to chemotherapy and survival was retrospectively analyzed in 28 patients with bulky retroperitoneal and disseminated seminoma treated between 1977 and 1983. The median age was 41 years (range: 23–52). All patients had histological evidence of pure testicular seminoma, however, 14 patients revealed moderate increases of human $\beta$-chorionic gonadotropin levels. Prior radiotherapy had been given to 9/28 (32%) patients. Treatment consisted of at least four courses of simultaneous or sequentially alternating therapy with cisplatin, vinblastine, bleomycin plus/minus adriamycin (PVB$\pm$A), administration of ifosfamide or combination therapy with ifosfamide/cisplatin (IFS/DDP) or ifosfamide/etoposide (IFS/ETP). Twenty-five of 28 patients (89%) achieved a complete (CR), and 3/28 patients a partial remission. Relapse occurred in 1/8 CR patients after adjuvant postchemotherapeutic irradiation, and in 1/11 patients without any further radiotherapy. So far, 23/28 patients (82%) are free of disease after a median follow-up of 28+ (14+→82+) months. Marked myelosuppression was observed in previously irradiated patients, mainly after PVB$\pm$A therapy. In two patients, transient nephrotoxicity developed after PVB and IFS/DDP, respectively. After PVB$\pm$A chemotherapy, three patients revealed polyneuropathy, paralytic subileus and bleomycin-induced pneumonitis, respectively. In conclusion, the present series suggests a high probability of continuous CR in even bulky retroperitoneal and widespread metastatic seminoma. So far, no definite conclusions can be made on the therapeutic superiority of one of the different chemotherapeutic regimens used. However, this preliminary experience suggests that the combination of ifosfamide and etoposide or cisplatin may prove less toxic than sequentially alternating or simultaneous PVB$\pm$A chemotherapy.

Seminomas, which account for ~40% (27–71%) of testicular cancers, generally tend to present as local (Stage I) or locoregional (Stage II) disease in 70–90% of the patients (Calman *et al.*, 1979; Dixon & Moore, 1952; Maier *et al.*, 1968; Mostofi, 1973; Thomas *et al.*, 1982). Orchiectomy and retroperitoneal radiotherapy in Stage I and minimal Stage II disease usually result in an excellent overall prognosis, with 5-year survival rates approaching 90%. However, long-term results have remained rather poor with up to 40–50% relapses when radiotherapy served as the sole treatment of bulky Stage II disease (Ball *et al.*, 1982; Smith *et al.*, 1979; Thomas *et al.*, 1982).

Besides some early trials reporting on the sensitivity of metastatic seminomas to single agent chemotherapy, recent studies, mostly utilizing cisplatin containing regimens originally employed for the treatment of nonseminomatous testicular cancer have described a 50–70% or even higher complete remission rate (Ball *et al.*, 1982; Mendenhall *et al.*, 1981; Vugrin & Whitmore, 1984). However, most series contained only a small number of patients, thereby precluding any definitive conclusion on the value of the regimens used. In addition, significant toxicity, predominantly occurring in previously irradiated patients, has been observed with most of these regimens.

Correspondence: J. Schuette

Received 31 August 1984; and in revised form 28 December 1984

Nevertheless, cytostatic chemotherapy appears to be the treatment of choice not only for widespread metastatic, but also for bulky retroperitoneal disease. In the present retrospective analysis, the efficacy and toxicity of different combination chemotherapies in the treatment of advanced disseminated seminomas has been studied.

### Materials and methods

### *Patients*

This report includes 28 patients with histologically confirmed pure seminoma of the testis who presented at our institution between 1977 and 1983. None of these patients had received prior chemotherapy. Patient characteristics are shown in Table I. The median age was 41 years (range: 23–52 years). Histological examination revealed a poorly differentiated (anaplastic) seminoma in 6 patients (21%) and a well differentiated (classical or typical) seminoma in 22 patients (79%). Fourteen patients (50%) showed a moderate elevation of human chorionic gonadotropin ($\beta$-hCG) serum levels prior to chemotherapy, which did not exceed $70\,mIU\,ml^{-1}$ (normal: $<5\,mIU\,ml^{-1}$) in 12/14 patients. Two patients demonstrated $\beta$-hCG levels of 150 and $210\,mIU\,ml^{-1}$ without any histological evidence of nonseminomatous tumour elements.

### *Pretreatment*

Patients were usually referred to our institution

© The Macmillan Press Ltd., 1985

**Table I** Patient characteristics

| | | |
|---|---|---|
| No. of evaluable pts | : 28 | |
| Median age | : 41 y | (range: 23–52) |
| Histology | | |
| poorly differentiated | : 6/28 | (21%) |
| well differentiated | : 22/28 | (79%) |
| Tumour marker | | |
| AFP elevation | : 0/28 | |
| β-HCG elevation | : 14/28 | (50%) |
| Pretreatment | | |
| Irradiation | : 9/28 | (32%) |
| retroperitoneal | : 5 | |
| retroperitoneal, mediastinal + supraclavicular | : 4 | |
| Laparotomy | : 7/28 | (25%) |

following the initial orchiectomy performed at other hospitals. Nine of the 28 patients (32%) had been previously treated with retroperitoneal ($n=5$), or retroperitoneal, mediastinal and supraclavicular ($n=4$) irradiation. Another 7 patients with Stage II C disease had undergone a prior laparotomy for diagnostic and staging purposes.

*Staging procedures*

Clinical staging generally included physical examination, chest X-rays, routine blood chemistry monitoring, abdominal sonograms, intravenous urography, liver and bone scans and lymphography when indicated, and, since 1979, computerized tomograms. In addition, serum was routinely collected for alpha-fetoprotein (AFP) and β-hCG determinations.

The Staging classification used has been originally developed for nonseminomatous testicular cancer (Seeber et al., 1980; Schuette et al., 1983):

—Stage II B: Retroperitoneal lymph node metastases, >2 and <5 cm in diameter.
—Stage II C: Bulky retroperitoneal disease, metastases >5 cm in maximum diameter, in most cases palpable, often with ureteral displacement.
—Stage III: Infra- and supradiaphragmatic lymph node metastases.
—Stage IV A: Minimal pulmonary with no measurable abdominal disease.
—Stage IV B: Advanced pulmonary (>10 $cm^3$) with no measurable abdominal disease.
—Stage IV C: Minimal pulmonary and abdominal (>2, <5 cm) disease.
—Stage IV D: Advanced pulmonary and abdominal disease (>5 cm) plus/minus extralymphatic metastases (liver, bone, brain, etc.).

One patient was classified as having Stage II B disease after previous laparotomy and an untraoperative macroscopic dissemination of viable tumour material; in addition, the β-hCG level was slightly elevated prior to chemotherapy. Fourteen patients presented with Stage II C, including 11/14 patients with maximum tumour diameters >10 cm. One patient had Stage III, and 4 patients Stage IV C disease. Stage IV D disease was recorded in 8 patients.

*Treatment schedules*

During the past 7 years different chemotherapeutic regimens have been used at our institution. From 1977–1980, chemotherapy of advanced metastatic seminomas closely resembled our treatment policy for nonseminomatous testicular tumours, consisting of the sequentially alternating administration of vinblastine/bleomycin and cisplatin/adriamycin in 9/28 patients (Niederle et al., 1983; Scheulen et al., 1980; Schuette et al., 1983). Treatment schedules and doses are shown in Table II. Two patients received the 3-drug combination of cisplatin, vinblastine and bleomycin (Einhorn & Donohue, 1979). Since 1980/81, mainly in the lights of some favourable results of early Phase II trials, chemotherapy of seminomas primarily consisted of ifosfamide, either alone or in combination with etoposide or cisplatin. Most patients received at least 4 courses (range: 4–9) of the fractionated chemotherapy with either regimen. Generally, treatment was repeated for at least 2 courses after the induction of complete remission.

**Table II** Treatment schedules (repeated every 3–4 weeks)

| | |
|---|---|
| I | Sequentially alternating chemotherapy, starting with either 2 courses of adriamycin/cisplatin or vinblastine/bleomycin. After 4 courses of chemotherapy, treatment was adapted to the individual needs. |
| | Adriamycin 60 mg $m^{-2}$ i.v., Day 1 |
| | Cisplatin 20 mg $m^{-2}$ i.v., Days 1–5 |
| | Vinblastine 0.15–0.2 mg $kg^{-1}$ i.v., Days 1+2 |
| | Bleomycin 30 mg i.v., Days 1–5 (continuous infusion) |
| II | Simultaneous 3-drug regimen: |
| | Cisplatin 20 mg $m^{-2}$ i.v., Days 1–5 |
| | Vinblastine 0.15 mg $kg^{-1}$ i.v., Days 1+2 |
| | Bleomycin 30 mg i.v., Days 1, 8+15 |
| III | Ifosfamide 60–80 mg $kg^{-1}$ i.v., Days 1–5 |
| IV | Ifosfamide 40–50 mg $kg^{-1}$ i.v., Days 1–5 |
| | Etoposide 100–120 mg$^{-2}$ i.v., Days 1, 3+5 |
| V | Ifosfamide 40–50 mg$^{-1}$ i.v., Days 1–5 |
| | Cisplatin 20 mg $m^{-2}$ i.v., Days 1–5 |

*Response criteria*

Tumour response was assessed before each treatment course. Objective response was classified according to standard criteria (Miller *et al.*, 1981): Complete remission (CR) was defined as the disappearance of all evaluable tumour parameters for at least one month. Partial remission (PR) was a more than 50% reduction of tumour volume.

### Results

### *Response*

All patients considered in this review were evaluable for response. Treatment results in relation to tumour stage are shown in Table III. The response rate was 100% with 25/28 patients (89%) achieving a complete, and 3/28 patients (11%) a partial remission.

Treatment results with regard to the different chemotherapeutic regimens used are given in Table IV. Complete remission was achieved in 7/7 patients treated with ifosfamide/cisplatin, and in 6/7 patients following ifosfamide/etoposide combination therapy. With the latter regimen one PR was attained in a patient after extensive prior irradiation. Ifosfamide alone resulted in 3/3 CR with one patient relapsing after 11 months. This patient was further salvaged by retroperitoneal irradiation. Sequentially alternating chemotherapy with cisplatin/adriamycin and vinblastine/bleomycin resulted in 8/9 CR. The simultaneous combination of cisplating, vinblastine and bleomycin (PVB) produced one complete and one partial remission.

Comparing the number of treatment cycles necessary for CR induction, sequentially alternating and PVB chemotherapy resulted in a median of 3, treatment with ifosfamide combination therapy in a median of 2 treatment courses.

Since experience with nonseminomatous testicular cancer suggested a useful role of adjuvant surgery for bulky retroperitoneal disease in increasing long-term survival, and $\beta$-hCG elevations might represent some nonseminomatous tumour elements in even "pure" seminomas, retroperitoneal lymph node dissection following the chemotherapeutic CR induction was performed in 6/14 $\beta$-hCG positive patients who initially presented with Stage II C disease. In each of these patients necrosis and dense fibrosis but no viable tumour residues were discovered by histological examination.

Overall, the outcome of treatment with regard to the presence of $\beta$-hCG increases is shown in Table V. Twelve of 14 $\beta$-hCG positive patients (86%) achieved CR with 10/14 patients still being alive and currently free of disease. One of the group of 4 treatment failures had Stage III, and 3/4 patients stage IV disease. On the other hand, 13/14 tumour marker negative patients (93%) attained CR. So far, all 14 patients are alive and free of disease,

**Table III** Response to chemotherapy by stage of seminoma

| *Stage* | *II B* (*n=1*) | *II C* (*n=14*) | *III* (*n=1*) | *IV C* (*n=4*) | *IV D* (*n=8*) | *Total* (*n=28*) |
|---|---|---|---|---|---|---|
| CR | 1 | 14 | 1 | 3 | 6 | 25 |
| Relapse | — | 1 | 1 | 1 | — | 3 |
| presently NED[a] | 1 | 14[b] | — | 2 | 6 | 23[b] |

NED: no evidence of disease.
[a]median follow-up: 28+ months.
[b]including one patient who attained a second CR following radiotherapy.

**Table IV:** Outcome of treatment by chemotherapy

| | *DDP/ADM* + *VLB/BLM* (*n=9*) | *DDP, VLB, BLM* (*n=2*) | *IFS* (*n=3*) | *IFS/DDP* (*n=7*) | *IFS/ETP* (*n=7*) |
|---|---|---|---|---|---|
| CR | 8 | 1 | 3 | 7 | 6 |
| PR | 1 | 1 | — | — | 1 |
| Relapse | 2 | — | 1 | — | — |
| presently NED | 6 | 1 | 3 | 7 | 6 |

NED: no evidence of disease (median follow-up 28+ months).
DDP: cisplatin, ADM: adriamycin, VLB: vinblastine.
BLM: bleomycin, IFS: ifosfamide, ETP: etoposide.

**Table V** Results of chemotherapy in relation to serum $\beta$-HCG levels ($n=28$)

| | *β-HCG elevated* ($n=14$) | *β-HCG not elevated* ($n=14$) |
|---|---|---|
| CR | 12 | 13 |
| presently NED | 10 | 13 |

including on PR patient who was salvaged with additional radiotherapy for residual retroperitoneal disease.

*Influence of radiotherapy on treatment results*

Complete remission was achieved in 7/9 previously irradiated patients (78%) and in 18/19 patients (95%) who had not received prior radiotherapy. Partial remission in both groups was observed only in Stage IV disease. Both previously irradiated PR patients had developed significant myelosuppression during chemotherapy requiring intensive supportive care, prolongation of treatment intervals and cytostatic dose reductions which might account for the treatment failure.

Eight patients with chemotherapy-induced CR were further allocated for an adjuvant irradiation of the primary advanced retroperitoneal (n=7) and/or mediastinal disease(n=3). One of these patients relapsed after 14 months and could not be salvaged by subsequent chemotherapy due to severe myelosuppression. In contrast, only one of 11 CR patients who did not receive prior radiotherapy or adjuvant irradiation after previous chemotherapy relapsed 11 months after ifosfamide and achieved a second CR following irradiation of recurrent retroperitoneal disease.

*Survival*

All patients are evaluable for a minimum follow-up of one year. Remission duration of CR patients ranges between 14+ and 82+ months (median: 28+), compared to 3, 10 and 15+ months in partial responders. Overall median survival is 28+ months with a median of 31+ months for complete responders (range: 15+→86+). So far, two PR and one CR patient died 12, 13 and 25 months after start of chemotherapy, respectively (Table IV).

*Toxicity*

Side effects usually included alopecia and myelosuppression (Table VI). Significant reductions of the standard cytostatic dosages were necessary in almost all patients after prior irradiation. Platelet transfusions and antibiotic treatment for severe thrombo- and leukocytopenia were required in one patient after PVB chemotherapy. Temporary increases of serum creatinine concentrations were observed in two patients after PVB and ifosfamide/cisplatin, respectively. Long-lasting neurotoxic side effects due to vinblastine (and cisplatin) became apparent in one patient after sequentially alternating chemotherapy with vinblastine/bleomycin and cisplatin/adriamycin. Interstitial lung fibrosis after a total dose of 300 mg of bleomycin occurred in a 43-year old patient who had previously received mediastinal irradiation. One patient developed a paralytic subileus after PVB. Nausea and vomiting usually occurred during cisplatin therapy but were significantly less intense and only

**Table VI** Toxicity of chemotherapy in metastatic seminoma

| *Type of side effect* | *DDP/ADM + VBL/BLM* | *DDP, VLB, BLM* | *IFS* | *IFS/DDP* | *IFS/ETP* |
|---|---|---|---|---|---|
| Leukocytopenia | | | | | |
| $<2000\,\mu l^{-1}$ | 2/6 | 2/2 | — | 3/6 | 1/5 |
| $<1000\,\mu l^{-1}$ | 1/6 | 1/2 | — | — | — |
| Thrombocytopenia | | | | | |
| $<100{,}000\,\mu l^{-1}$ | 1/6 | 2/2 | — | 1/6 | — |
| $<50{,}000\,\mu l^{-1}$ | 1/6 | 1/2 | — | — | — |
| Alopecia | 9/9 | 2/2 | 3/3 | 7/7 | 7/7 |
| Nephrotoxicity (creatinine > 1.2 mg%) | — | 1/2 | — | 1/7 | — |
| Polyneuropathy | — | 1/2 | — | — | — |
| Pneumonitis/Lung fibrosis | 1/9 | — | — | — | — |
| Paralytic subileus | — | 1/2 | — | — | — |
| Haematuria | — | — | — | — | — |

infrequently recorded with ifosfamide and ifosfamide/etoposide therapy.

### Discussion

Since the original report by Blokhin *et al.* (1958) demonstrating activity of cytostatic chemotherapy in disseminated testicular seminoma, recent reports of the use of single agent and combination chemotherapy have shown encouraging results suggesting an at least similar chemosensitivity of disseminated seminoma as compared to nonseminomatous testicular cancer (Blokhin *et al.*, 1958; Chebotareva, 1964; Golbey, 1970; Mackenzie, 1966; Monfardini *et al.*, 1972; Vugrin *et al.*, 1981; Whitmore *et al.*, 1977). However, since only 5–15% of patients initially present with advanced locoregional or disseminated disease and only few patients relapse after prior radiotherapy of minimal retroperitoneal disease, most trials include only small numbers of patients (Table VII). So far, chemotherapy with cisplatin, vinblastine and bleomycin (PVB) has been the most widely used combination resulting in a 50–70% CR rate with most CR patients remaining free of disease (Einhorn & Williams, 1980). Nevertheless, significant morbidity preferably occurring after prior radiotherapy has been observed by several authors (Ball *et al.*, 1982; Mendenhall *et al.*, 1981; Wajsman *et al.*, 1983).

The present series including 28 patients with widespread metastatic and/or advanced retroperitoneal seminoma has demonstrated an excellent chemosensitivity with 23/28 (82%) patients being currently free of disease after a median follow-up of 28+ months. Thus, in comparison to the 30–50% actual long-term survival rates (median follow-up ≧ 2 years) described for patients with similar stages of advanced and bulky nonseminomatous testicular cancer, disseminated seminoma in our experience appears to be the germ cell cancer most responsive to cytostatic chemotherapy (Einhorn & Donohue, 1979; Niederle *et al.*, 1983; Vugrin *et al.*, 1982). At present, no substantial data are available to suggest therapeutic benefit from consolidating radiotherapy in complete responders. In this series, relapse occurred in one of 8 patients with and in one of 11 patients without additional irradiation.

Similar to other reports, adjuvant retroperitoneal lymph node dissection after chemotherapy as carried out in 6/14 of the initially $\beta$-hCG positive, bulky disease patients might be of only limited value since microscopic tumour residues were discovered in none of these patients (Vugrin & Whitmore, 1984; Samuels *et al.*, 1980). In addition, complete lymphadenectomy was difficult to perform because of the apparently dense fibrous tissue found in the retroperitoneal area. Surgical complications, however, did not occur.

In accordance with the results of several radiotherapeutic trials, moderate elevation of $\beta$-hCG levels, although suggesting the presence of nonseminomatous, chorionic tumour elements, did not seem, in our patients to adversely influence the response and survival rates as compared to serologically negative patients (Ball *et al.*, 1982; Javadpour *et al.*, 1978; Mauch *et al.*, 1979). The treatment failures observed in $\beta$-hCG positive patients might be due to the more advanced stage of disease and/or prior irradiation.

In conclusion, the present series substantiates the role of combination chemotherapy in advanced

**Table VII** Treatment results with chemotherapy in metastatic seminoma (selected literature)

| *Treatment schedule* | *No. pts* | *CR* (%) | *Long-term survival* (%) | *Author (see under References)* |
|---|---|---|---|---|
| VLB, BLM | 11 | 4 (36) | 2 (18) | Samuels *et al.* (1980) |
| DDP, VCR, BLM, PRED or DDP, ETP | 12 | 12 (100) | 12 (100) | Wajsman *et al.* (1983) |
| BLM, CTX, VCR, MTX, 5-FU | 18 | 10 (55) | 6 (33) | Samuels *et al.* (1980) |
| DDP, VLB, BLM | 8 | 4 (50) | 8 (100)[a] | Ball *et al.* (1982) |
| CTX, DDP | 9 | 5 (55) | 2 (22) | Vugrin & Whitmore (1984) |
| CTX, DDP, BLM, VLB, Act D (VAB-6) | 7 | 7 (100) | 5 (71) | Vugrin & Whitemore (1984) |
| DDP, VLB, BLM+ADM | 19 | 12 (63) | 11 (58) | Einhorn & Williams (1980) |
| Present series | 28 | 25 (89) | 23 (82) | |

[a]Results obtained following radiotherapy in partial responders.
DDP: cisplatin, BLM: bleomycin, VLB: vinblastine, ADM: adriamycin, CTX: cyclophosphamide, ETP: etoposide, MTX: methotrexate, 5-FU: 5-fluorouracil, ActD: actinomycin D, PRED: prednisone, VCR: vincristine.

seminoma. Since 80–90% of all relapses have been shown to occur within 2–3 years after remission induction, most of our complete responders probably might attain continuous CR (Calman *et al.*, 1979; Thomas *et al.*, 1982). However, with respect to the limited number of patients and the retrospective design of this analysis, no definite conclusions can yet be made on the therapeutic superiority of any one of the different chemotherapeutic regimens used. Nevertheless, this preliminary experience suggests that the combination of ifosfamide and etoposide or cisplatin may prove equally effective and, most importantly, less toxic as compared to the conventional simultaneous or sequentially alternating PVB$\pm$A chemotherapy.

### References

BALL, D., BARRETT, A. & PECKHAM, M.J. (1982). The management of metastatic seminoma testis. *Cancer*, **50**, 2289.

BLOKHIN, N., LARIONOV, L., PEREVODCHIKOVA, N., CHEBOTAREVA, L. & MERKULOVA, N. (1958). Clinical experience with sarcolysin in neoplastic disease. *Ann. N.Y. Acad. Sci.*, **68**, 1128.

CALMAN, F.M.B., PECKHAM, M.J. & HENDRY, W.F. (1979). The pattern of spread and treatment of metastases in testicular seminoma. *Br. J. Urol.*, **51**, 154.

CHEBOTAREVA, L.I. (1964). Late results of sarcolysin therapy in tumours of the testes. *Acta Un. Int. Can.*, **20**, 380.

DIXON, F.J. & MOORE, R.A. (1952). Tumors of the male sex organs. *Atlas of Tumor Pathology, Sect. VIII*, Fasc. 31b/32. Washington, D.C., Armed Forces Institute of Pathology.

EINHORN, L.H. & DONOHUE, J.P. (1979). Combination chemotherapy in disseminated testicular cancer: The Indiana University experience. *Semin. Oncol.*, **6**, 87.

EINHORN, L.H. & WILLIAMS, S.D. (1980). Chemotherapy of disseminated seminoma. *Cancer Clin. Trials*, **3**, 307.

GOLBEY, R.B. (1970). The place of chemotherapy in the treatment of testicular tumors. *JAMA*, **213**, 101.

JAVADPOUR, N., McINTIRE, K.R. & WALDMANN, T.A. (1978). Human chorionic gonadotropin (HCG) and alpha-feto-protein (AFP) in sera and tumour cells of patients with testicular seminoma: A prospective study. *Cancer*, **42**, 2768.

MACKENZIE, A.R. (1966). The chemotherapy of metastatic seminoma. *J. Urol.*, **96**, 790.

MAIER, J.G., MITTEMEYER, B.T. & SULAK, M.H. (1968). Treatment and prognosis in seminoma of the testis. *J. Urol.*, **99**, 72.

MAUCH, P., WEICHSELBAUM, R. & BOTNICK, L. (1979). The significance of positive chorionic gonadotropins in apparently pure seminoma of the testis. *Int. J. Radiat. Oncol. Biol. Phys.*, **5**, 887.

MENDENHALL, W.L., WILLIAMS, S.D., EINHORN, L.H. & DONOHUE, J.P. (1981). Disseminated seminoma: re-evaluation of treatment protocols. *J. Urol.*, **126**, 493.

MILLER, A.B., HOOGSTRATEN, B., STAQUET, M. & WINKLER, A. (1981). Reporting results of cancer treatment. *Cancer*, **47**, 207.

MONFARDINI, S., BAJETTA, E., MUSUMECI, R. & BONADONNA, G. (1972). Clinical use of adriamycin in advanced testicular cancer. *J. Urol.*, **108**, 293.

MOSTOFI, F.K. (1973). Testicular tumors: Epidemiologic, etiologic, and pathologic features. *Cancer*, **32**, 1186.

NIEDERLE, N., SCHUETTE, J., KRISCHKE, W. & 4 others. (1983). Alternating combination chemotherapy in disseminated testicular testicular cancer. 2nd European Conference on Clinical Oncology and Cancer Nursing, Amsterdam, November 2–5, p. 151.

SAMUELS, M., LOGOTHETIS, C., TRINDADE, A. & JOHNSON, D.E. (1980). Sequential weekly pulse-dose cis-platinum for far-advanced seminoma. *Proc. Am. Assoc. Cancer Res. & Am. Soc. Clin. Oncol.*, **21**, 423.

SCHEULEN, M.E., SEEBER, S., SCHILCHER, R.B., MEIER, C.R. & SCHMIDT, C.G. (1980). Sequential combination chemotherapy with vinblastine-bleomycin and doxorubicin-cis-dichlorodiammineplatinum(II) in disseminated nonseminomatous testicular cancer. *Cancer Treat. Reap.*, **64**, 599.

SEEBER, S., SCHEULEN, M.E., SCHILCHER, R.B. & 5 others. (1980). Sequential combination chemotherapy with vinblastine-bleomycin and adriamycin-cisplatin in early and late testicular cancer. In: *Cisplatin: Current Status and New Developments*. (Eds. Prestayko *et al.*) Academic Press, New York, London, p. 329.

SCHUETTE, J., BREMER, K., NIEDERLE, N., SCHOETENSACK, B., SCHMIDT, C.G. & SEEBER, S. (1983). Sequentiell-alternierende Chemotherapie nichtseminomatöser Hodentumoren mit Adriamycin/Cisplatin und Bleomycin/Vinblastin: Therapieansprechen und versagen in Abhängigkeit von Histologie und Tumorstadium. *Onkologie*, **6**, 16.

SMITH, R.B., DEKERNION, J.B. & SKINNER, D.G. (1979). Management of advanced testicular seminoma. *J. Urol.*, **121**, 429.

THOMAS, G.M., RIDER, W.D., DEMBO, A.J. & 5 others. (1982). Seminoma of the testis: Results of treatment and patterns of failure after radiation therapy. *Int. J. Radiat. Oncol. Biol. Phys.*, **8**, 165.

VUGRIN, D., WHITMORE, W., OCHOA, M. & GOLBEY, R. (1981). Cis-diammine-dichloroplatinum(II) (CDDP) in combination chemotherapy of metastatic seminoma. *Proc. Am. Assoc. Cancer Res.*, **22**, 166.

VUGRIN, D., WHITMORE, W.F. & GOLBEY, R.B. (1982). Effect of shorter induction intervals on complete remission (CR) rates in advanced nonseminomatous germ cell tumours of the testis (NSGCTT). *Proc. Am. Assoc. Cancer Res.*, **23**, 148.

VUGRIN, D. & WHITMORE, W.F. (1984). The VAB-6 regimen in the treatment of metastatic seminoma. *Cancer*, **53**, 2422.

WAJSMAN, Z., BECKLEY, S.A. & PONTES, J.E. (1983). Changing concepts in the treatment of advanced seminomatous tumours. *J. Urol.*, **129**, 303.

WHITMORE, W.F., SMITH, A., YAGODA, A., CVITKOVIC, S. & GOLBEY, R. (1977). Chemotherapy of seminoma. Tumors of the male genital system. *Recent Results Cancer Res.*, **60**, 244.